# Societal costs and quality of life analysis in patients undergoing resective epilepsy surgery: A one-year follow-up

**DOI:** 10.1016/j.ebr.2023.100635

**Published:** 2023-11-19

**Authors:** L. Maas, J. Kellenaers, G. van Mastrigt, S.M.J van Kuijk, M.C.G. Vlooswijk, M. Hiligsmann, S. Klinkenberg, L. Wagner, J. Nelissen, O.E.M.G. Schijns, H.J.M. Majoie, K. Rijkers

**Affiliations:** a Department of Neurosurgery, Maastricht University Medical Center, Maastricht, The Netherlands; b Department of Health Services Research, CAPHRI Care and Public Health Research Institute, Maastricht University, The Netherlands; c Department of Clinical Epidemiology and Medical Technology Assessment, Maastricht University Medical Center, The Netherlands; d School for Mental Health & Neuroscience, University Maastricht, Maastricht, The Netherlands; e ACE: Academic Center for Epileptology, Maastricht Heeze, The Netherlands; f Department of Neurology, Maastricht University Medical Center, The Netherlands

**Keywords:** Resective epilepsy surgery, Societal costs, Cost-effectiveness, Epilepsy, Drug-resistant epilepsy patients, Quality of life

## Abstract

Although effectiveness of Resective Epilepsy Surgery (RES) for patients with drug-resistant epilepsy (DRE) is widely proven, research on the impact of societal costs (SC) is lacking. The aim of this study is to provide both clinical and economic outcomes of RES by offering an overview of treatment effectiveness as well as SC of RES in a cohort of 30 Dutch DRE patients. This project serves as a pilot project to offer an up-to-date model for larger cost-effectiveness studies. Medical consumption, productivity losses, disease-specific and generic health-related quality of life (QoL), and seizure frequency were assessed before and 3-, 6-, and 12-months post-surgery with validated questionnaires. Linear mixed models, ANOVAs, and logistic regressions were performed. SC for the first year after RES entailed €54,376 and decreased over time. Moreover, 50% of patients experienced a clinically important increase in disease-specific QoL and 53% of patients in generic health-related QoL. Lastly, 73% of patients reached seizure freedom 12 months postoperative. Seizure reduction was correlated with increase in disease-specific QoL. Within one year after surgery, RES leads to reduction in SC and improvements in QoL over time. Future research should encompass longer follow-up periods, larger sample size, and a cost-effectiveness analysis with a comparator.

## Highlights

• Societal costs decrease over the first year after resective epilepsy surgery.
• Quality of life increases after resective epilepsy surgery.
• Seizure freedom is reached in 73% of cases one year after resective epilepsy surgery.

## Background

Epilepsy reduces Quality of life (QoL) and increases the emotional, physical, and financial burden on patients with epilepsy and society considerably. The extent of the burden depends on aspects such as seizure type and response to anti-seizure medication (ASM) [1]. Epilepsy affects around 50 million people worldwide [2]. In the Netherlands, fifty-five per 100,000 people are diagnosed with epilepsy annually, which amounts to 5,000–10,000 new patients annually [3], [4], [5]. Twenty to forty percent of epilepsy patients will develop drug resistance and about 20–50% of these drug-resistant epilepsy (DRE) patients are eligible for Resective Epilepsy Surgery (RES) [6], [7], [8], [9], [10]. RES has shown great potential for treatment of DRE patients and may offer a solution for (a) those who continue to circulate as patients in the healthcare system due to their drug-resistance and (b) the costly care-as-usual of DRE patients of €20,751 a year and its economic effect on society [11], [12], [13], [14], [15], [16]. In order to calculate the effect of DRE on societal costs (SC) of epilepsy patients, data are needed, but are scarcely available. Data that are necessary for conducting cost-effectiveness analyses encompasses clinical outcomes such as QoL and Quality Adjusted Life Years (QALY) data, in addition to economic outcomes, which comprise both direct (healthcare) and indirect (non-healthcare) costs.

Widely available are data on the clinical effectiveness of RES, with 50–68% of patients becoming seizure free (for a Cochrane review, see [17]). In addition, various studies have reported on QoL of RES in epilepsy, including study designs comparing pre- and post-operative QoL, randomized clinical trials [14], [18], [19], and retrospective and prospective cohort studies with ASM control groups, suggesting a significant QoL increase after surgery and this increase is related to seizure reduction [18], [20], [21], [22].

In contrast, few papers have been published focusing on costs or cost-effectiveness of RES. Choi et al. and Sheik et al. presented a cost-effectiveness analysis of RES using decision analytic models [23], [24]. A study conducted in France collected real-world data from 15 centers over 2 years and demonstrated cost-effectiveness of RES between 9 and 10 years after surgery [25]. Another American cost study reported that annually RES is $6,800 lower than care-as-usual and demonstrated RES to be cost-saving after 4 years [26]. Although these trial-based studies present cost-effectiveness of RES on real-world data, none of the studies have incorporated indirect cost valuation to determine their cost-effectiveness. Furthermore, these economic studies did not use a societal perspective and few included both costs and disease-specific QoL data in the same study.

Therefore, the aim of this study was to provide an analysis that covers both economic and disease-specific clinical outcomes by offering an overview of the societal (i.e., direct and indirect) costs directly related to RES. The costs of diagnostic/presurgical evaluation necessary for identifying patients as suitable candidates for resective brain surgery (including neuropsychological evaluation, diagnostic Magnetic Resonance Imaging (MRI), video-electroencephalography (EEG), positron emission tomography (PET), single-photon emission computed tomography (SPECT), magnetoencephalography (MEG), and stereo-EEG) are not included in this analysis. This project serves as a pilot project to offer an up-to-date model for larger cost-effectiveness studies. We hypothesize that (i) SC decrease within the first year after RES; (ii) RES enhances QoL to a clinically important degree and reduced seizure frequency in DRE patients in the first year after surgery; (iii) patients with more comorbidities would benefit less from RES compared to patients with less comorbidities; and (iv) seizure reduction subsequently leads to an increase in QoL in DRE patients in the first year after RES.

## Materials & methods

A single center prospective cohort study was conducted. A cohort study design was chosen due to the small population size of the Netherlands and recent failures surrounding inclusion of sufficient patients in randomized controlled trials in larger countries such as the United States [11]. This study was approved by the Medical Ethics Review Committee (METC) of Maastricht UMC+ (2019-1134) and registered at the Dutch Trial Register as RESQUE study (Resective Epilepsy Surgery, Quality of life and Economic evaluation) (NL8278). The STROBE and CHEERS checklists were used to strengthen the reporting of our study (Supplementary Table 1 & 2) [27], [28].

### Study population

All Dutch-speaking DRE patients equal to or over the age of 16 who were referred to the Department of Neurosurgery of the Academic Center for Epileptology, Maastricht and Heeze (ACE) for RES between January 2019 and December 2022 were deemed eligible. To measure a Minimal Clinically Important Change (MCIC) in disease-specific QoL with a power of 80%, at least 25 patients had to be included [29]. People with a total intelligence quotient (TIQ) score of < 70 were excluded, which was determined through a neuropsychological assessment by a neuropsychologist. In case of disharmonic intelligence profiles (i.e., discrepancy between verbal and non-verbal/visual-spatial TIQ), the verbal TIQ was leading.

### Questionnaires

Participants were asked to complete standardized validated questionnaires two weeks before surgery, and 3-, 6-, and 12-months after surgery. The questionnaires referred to the previous 3 months prior to the time of questionnaire completion. The primary outcome was the change in SC. Secondary outcomes were (i) disease-specific QoL measure by the Quality of Life in Epilepsy 31P (QOLIE-31P) questionnaire [30] (ii) generic health-related QoL measured by (European Quality of Life 5 Dimension (EQ-5D-5L)) [31], and (iii) seizure outcome according to the International League Against Epilepsy (ILAE) outcome scale [32].

### Costs

To calculate SC, validated questionnaires for medical consumption (using iMTA Medical Consumption Questionnaire iMCQ) [33] and productivity losses (using iMTA Productivity Cost Questionnaire iPCQ) [34] were used to collect data 3 months before surgery, and then were administered at 3, 6, and 12 months after surgery. The answers to these questionnaires were supplemented with their corresponding patient files from SAP General User Interface for Windows 7.70 (SAP GUI 770) where necessary. In the context of our study, it is important to acknowledge the disparity between the diagnostic/presurgical workup phases and medical consumption immediately prior to RES. The former frequently extends over several months to even years and refers to medical consumption related to identifying DRE and eligibility of those DRE patients for RES. The latter primarily occurs in the immediate time frame prior to RES and refers to all medical consumption directly related to RES of patients already diagnosed with DRE and deemed eligible for RES. Given this distinction and alignment of our objective on reporting costs of RES with the validated questionnaires, the chosen 3-month timeframe before surgery was expected to provide adequate information for this study. A societal perspective was chosen in line with the Dutch guideline for economic evaluations, as it provides a broader scope of cost analyses in comparison with a healthcare perspective that only incorporates direct medical costs [35]. This broader scope entails the use of resources outside the healthcare sector, e.g. productivity losses, which may also impact society.

Cost valuation was done using the Dutch guideline for direct (healthcare) and indirect (non-healthcare) costs for one year post surgery [36]. All costs are reported in euros. Discounting (i.e., converting future costs to relative value) was not performed as the follow-up period did not exceed one year. Travel costs were calculated by multiplying the average distance in kilometers with standard price including parking costs if applicable. Supplementary Table 3 provides an overview of all reference prices per cost category.

### Data collection

Patients received an information letter including the informed consent form by mail or email, along with their invitation for their first neurosurgical consultation. If patients were willing to participate, they either sent their informed consent form back after reading and signing it or agreed to participate and gave informed consent after contact with the researcher during the outpatient clinic visit.

Patients received their survey package timely by e-mail via Castor Electronic Data Capture (EDC). Additional demographic and clinical data were collected using patient files from SAP GUI 770, i.e.: age, date of surgery, sex, body mass index (BMI), hemisphere side of surgery, type of surgery, surgery complications, comorbidities, previous resection, previous neuromodulation, history of ketogenic diet, age of epilepsy onset, number of ASM currently using, and total number of ASM used in the past (simultaneously or consequently). Duration of epilepsy was calculated by subtracting patients’ age at onset from their current age.

### Valuation

Medical consumption and productivity costs were calculated for the first year after RES along with these costs 3 months before the surgery in order to include costs directly related to surgery, e.g. neurosurgical and anesthesia consultation and MRI-scanning of the brain used for intraoperative neuronavigation. Costs were divided into direct costs (including medication, general practitioner, practice nurse, social worker, physiotherapist, occupational therapist, speech therapist, dietician, homeopath, psychologist, occupational physician, emergency department, ambulance, diagnostics such as MRI scans, specialist or epilepsy nurse consultations, hospital or epilepsy centre admissions) and indirect costs (including informal care by family or friends, paid home care, unpaid labor absenteeism, paid work absenteeism). Direct and indirect costs make up the SC. Due to the sensitive nature of the hospital’s financial information, costs of the surgery itself were not available, and instead were calculated by multiplying the cost of surgery per minute by the average operation duration in minutes. The cost per minute was provided by the financial control department of ACE and consists of the amount of money attributed to using a fully equipped and staffed neurosurgical operating room, with specific hardware available, e.g. microscope and navigation equipment. Direct and indirect costs were based on 2014 reference values [37], [38] and were adjusted to 2022 with data from the Centraal Bureau van de Statistiek (CBS) [39]. The friction cost method was used to calculate loss of paid and unpaid work [36]. For unemployed or retired patients or students, loss of paid work was not included, however their daily occupation was recognized as unpaid work.

### Statistical analysis

For baseline characteristics, continuous variables were represented as mean and standard deviation (SD). Categorical variables are presented as frequencies and percentages.

To account for missing data, multiple imputation was used according to the method described by Van Buuren et al. [40], [41]. Thirty imputed datasets were created and predictive mean matching was used for continuous, polytomous regression for categorical, and logistic regression for dichotomous variables. Prior to analysis, total and subscores were calculated for disease-specific QoL scores and generic health-related QoL utility scores were calculated for generic health-related QoL based on the Dutch Valuation set [37], [38]. Seizure frequency was dichotomized for statistical testing into a low (ILAE class 1–2) and high (ILAE class 3–5) seizure frequency group. A MCIC was validated and considered to be 11.8 points for disease-specific QoL total scores [11] and half the standard deviation of the sample for generic health-related QoL utility scores [42].

Linear mixed-effects models were used to analyze (i) change of QoL measures and seizure frequency over time; and (ii) the association between disease-specific or generic health-related QoL with demographic variables (i.e.: age, date of surgery, sex, BMI, hemisphere side of surgery, type of surgery, surgery complications, comorbidities, previous resection, previous neuromodulation, history of ketogenic diet, age of epilepsy onset, number of ASM currently using, and total number of ASM used in the past). To determine the association between seizure frequency and QoL, we used linear regression. Where applicable, the best model fit was determined using the Akaike Information Criterion (AIC) which would later be used in the multivariable analyses [43].

Results from linear mixed-effects models were presented as coefficient with its 95% confidence interval (CI) and p-value. Results from logistic regressions were presented as odds ratio, including 95% CI and p-value. When presenting MCIC QoL scores, non-responders referred to patients who decreased in their disease-specific QoL scores after surgery, intermediate responders referred to patients who increased in their disease-specific QoL scores after surgery but not as much as the MCIC, and responders referred to patients who reached a MCIC increase or more in their disease-specific QoL scores after surgery. For seizure frequency, responders were classified as patient who reached ILAE class 1 or 2, intermediate responders were classified as patients who reached ILAE class 3 or 4, and non-responders were classified as patients who reached ILAE 5 or 6, twelve months after surgery.

## Results

### Patients’ characteristics

Thirty DRE patients who were deemed eligible for RES were included. Patient characteristics are shown in Table 1. There were 3.3–17.5% missing data per variable (Supplementary Fig. 1). Information on the current number of ASM was not retrievable for 3 patients. ASM history was not retrievable for one patient.

**Table 1:** Demographics and patient characteristics for each patient. Continuous variables are presented by the mean and standard deviation (SD) and binomial variables are presented in frequencies (freq), categorical variables with more than two categories are presented as frequencies and percentages. ATL, anterior temporal lobectomy; AH, amygdalohippocampectomy. BMI, body mass index; Nr, number; ASM, anti-seizure medication, NA, not applicable.

Characteristics	Findings (n = 30)
Age, years (mean +/− SD)	44 +/− 14.3
Sex (female)	10 (33%)
BMI, kg/m^2^ (mean +/− SD)	25.5 +/− 4.8
Education (range)	
Elementary school	2 (7%)
Lower vocational education	2 (7%)
Pre-vocational secondary education	2 (7%)
Secondary vocational education	7 (23%)
Senior general secondary/pre-university education	4 (13%)
Higher professional education	6 (20%)
University education	6 (20%)
Other	1 (3%)
Employment status	
Employed	17 (57%)
Student	2 (6%)
Unemployed	11 (37%)
Age onset, years (mean +/− SD)	25 +/− 16.4
Duration, years (mean +/− SD)	16.2 +/− 12.9
Pre-operative seizure frequency	
Seizure free	0
Only auras	0
1-3 seizure days per year	0
4-12 seizure days per year	4 (13%)
Daily seizures	25 (84%)
NA	1 (3%)
Operation in dominant hemisphere (freq)	15 (50%)
type surgery	
Temporal	28 (93%)
Extra-temporal	2 (7%)
Type temporal surgery (freq)	
ATL with AH	14 (50%)
ATL without AH	1 (3%)
Selective AH	12 (44%)
Resection of temporal lesion	1 (3%)
Surgery complications	2 (6%)
OR history (freq)	4 (13%)
Comorbidities (freq)	12 (40%)
Neuromodulation via Nervus Vagus Stimulation (freq)	2 (6%)
Ketogenic diet (freq)	1 (3%)
NR of current ASM (freq)	
1–2	6 (20%)
3–4	15 (50%)
5+	6 (20%)
NA	3 (10%)
ASM history (freq)	
1–2	1 (3%)
3–4	16 (54%)
5+	12 (40%)
NA	1 (3%)

Eighteen patients did not suffer from comorbidities. The remainder of the patients suffered from a combination of several diseases (basilar artery aneurysm, endometriosis, hypertension, vascular encephalopathy, hyperthyroidism, diabetes, leukemia, meningoencephalitis, osteoporosis, dyspepsia, depression, irritable bowel syndrome (IBS), orthopnea, addiction, transient ischemic attack (TIA), and/or heart attack). In the 3 months prior to RES (temporal or extra-temporal, see Table 1 for types of resection), none of the patients received subdural grids or sEEG evaluation as part of their diagnostic evaluation.

### Societal costs

**Fig. 1:**
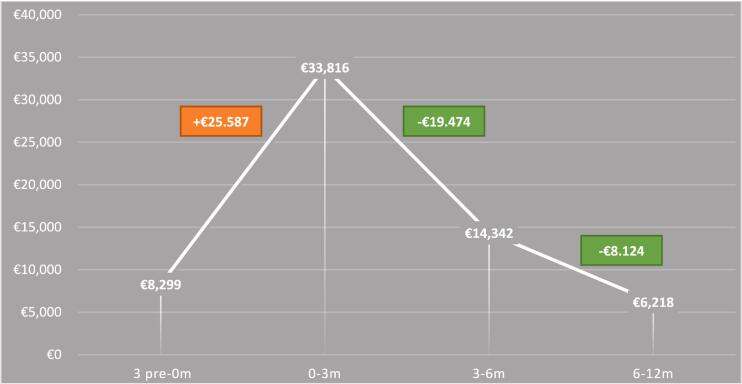
Total societal costs per time point, in red (increase in costs) and green (decrease in costs) boxes the differences in costs between time points are illustrated. (For interpretation of the references to colour in this figure legend, the reader is referred to the web version of this article.)

The SC of the first year after RES were €54,376. These costs included surgery costs and all direct and indirect costs, such as hospitalization days, consultations, transportation to hospital, etc. Considering the average costs of one minute of a neurosurgical operation and calculating the average duration of RES procedure, the average costs of surgery were €3,545. Table 2 and Fig. 1 illustrate the SC per time point, along with the difference in costs. Sixty-nine percent of non-employed patients suffered from comorbidities compared to 29% of the employed patients (Supplementary Table 4). There were no changes in individual employment status over time.

**Table 2:** Average imputed resource use and costs (euros) per time point of the sample (n = 30). Calculated by the friction cost methods. Diagnostic treatments includes MRI, PET, CT scans, blood work, etc.

**Unit**		**3 months pre-0 months pre-surgery**					**0-3 months post-surgery**					**3-6 months post-surgery**					**9-12 months post-surgery**					Mean differences*			**Total**	**Total**
**Per patient**		**Resource use**		**Cost**			**Resource use**		**Cost**			**Resource use**		**Cost**			**Resource use**		**Cost**				**Months**		**total**	
		**Mean**	**SD**	**Mean**	**SD**	**Median**	**Mean**	**SD**	**Mean**	**SD**	**Median**	**Mean**	**SD**	**Mean**	**SD**	**Median**	**Mean**	**SD**	**Mean**	**SD**	**Median**	**-3-0 & 0-3**	**0-3 & 3-6m**	**3-6 & 9-12m**	**0-12m**	**pre surg - 12m**
					**Healthcare costs**																					
GP	Contact	0.5	1.17	€ 20	€ 47	€ 0	0.9	0.95	€ 37	€ 35	€ 40	0.7	0.88	€ 28	€ 35	€ 0	1	1,50	€ 39	€ 60	€ 0	€ 17	−€9	€ 11	€ 142	€ 162
POH	Contact	0.3	0.82	€ 7	€ 17	€ 0	0.3	1.11	€ 6	€ 23	€ 0	0.5	2.50	€ 11	€ 52	€ 0	0,6	1,47	€ 13	€ 30	€ 0	−€1	€ 5	€ 2	€ 44	€ 50
Social worker	Contact	0.5	2.56	€ 40	€ 203	€ 0	0.1	0.46	€ 10	€ 36	€ 0	0.3	0.90	€ 20	€ 71	€ 0	0,1	0,21	€ 4	€ 17	€ 0	−€29	€ 10	−€16	€ 38	€ 77
Physio	Contact	1.3	3.67	€ 52	€ 148	€ 0	1.3	3.21	€ 51	€ 129	€ 0	1.6	3.97	€ 66	€ 160	0	1,1	2,70	€ 42	€ 109	€ 0	−€2	€ 15	−€23	€ 201	€ 253
Occ	contact	0.3	0.18	€ 2	€ 7	€ 0	0.1	0.21	€ 2	€ 8	€ 0	0.3	1.22	€ 10	€ 49	€ 0	0	0	€ 0	€ 0	€ 0	€ 1	€ 8	−€10	€ 12	€ 13
Speech	contact	0.2	0.91	€ 6	€ 34	€ 0	0	0	€ 0	€ 0	€ 0	0	0	€ 0	€ 0	€ 0	0,4	1,92	€ 15	€ 70	€ 0	−€6	€ 0	€ 15	€ 30	€ 36
Dietician	contact	0.1	0.40	€ 7	€ 29	€ 0	0.1	0.42	€ 6	€ 30	€ 0	0	0	€ 0	€ 0	€ 0	0	0	€ 0	€ 0	€ 0	−€1	−€6	€ 0	€ 6	€ 14
Homeo	Contact	0	0	€ 0	€ 0	€ 0	0.1	0.21	€ 5	€ 22	€ 0	0.1	0.20	€ 5	€ 21	€ 0	0	0	€ 0	€ 0	€ 0	€ 5	−€1	−€4	€ 9	€ 9
Psycho	Contact	1	2.27	€ 115	€ 261	€ 0	0.4	0.99	€ 45	€ 113	€ 0	0.4	1.02	€ 48	€ 117	€ 0	0,4	0,90	€ 42	€ 104	€ 0	−€70	€ 3	−€6	€ 176	€ 291
Occupational physician	Contact	0.3	0.94	€ 90	€ 320	€ 0	0.9	1.01	€ 295	€ 343	€ 339	0.7	1.04	€ 240	€ 353	€ 0	0,7	1,04	€ 231	€ 353	€ 0	€ 204	−€55	−€9	€ 997	€ 1.087
SEH	Contact	0	0	€ 0	€ 0	€ 0	0.1	0.34	€ 41	€ 109	€ 0	0.1	0.41	€ 26	€ 129	€ 0	0,1	0,21	€ 14	€ 68	€ 0	€ 41	−€15	−€12	€ 97	€ 97
Ambulance	contact	0.1	0.40	€ 63	€ 253	€ 0	0.1	0.29	€ 55	€ 181	€ 0	0.1	0.41	€ 52	€ 257	€ 0	0	0	€ 0	€ 0	€ 0	−€8	−€2	−€52	€ 107	€ 170
Diagnostic treatment	Treatment	1.6	0.90	€ 364	€ 178	€ 252	0.8	0.73	€ 180	€ 170	€ 252	0.3	0.66	€ 75	€ 134	€ 0	0,3	0,47	€ 77	€ 118	€ 0	−€184	−€105	€ 2	€ 410	€ 774
Epileptic institution	Visit	0.1	0.18	€ 6	€ 34	€ 0	0.1	0.35	€ 26	€ 66	€ 0	0	0	€ 0	€ 0	€ 0	0,1	0,64	€ 26	€ 120	€ 0	€ 19	−€26	€ 26	€ 77	€ 83
Epileptic institution	Night	0.1	0.18	€ 19	€ 103	€ 0	0.1	0.34	€ 73	€ 194	€ 0	0	0	€ 0	€ 0	€ 0	0,1	0,21	€ 26	€ 120	€ 0	€ 55	−€73	€ 26	€ 124	€ 14
Hospital	hospitalization	3.9	3.59	€ 758	€ 3	€ 0	3.6	4.59	€ 6,273	€ 4,436	€ 3,545	1.8	2.05	€ 556	€ 1,914	€ 0	2	2	€ 187	€ 856	€ 0	€ 1,970	−€2,172	−€369	€ 7,203	€ 7,961
Medication	-	-	-	€ 2,123	€ 6,036	€ 401	-	-	€ 633	€ 1,376	€ 232	-	-	€ 467	€ 426	€ 342	-	-	€ 325	€ 454	€ 77	−€1,490	−€166	−€142	€ 1,750	€ 3,873

**Total healthcare costs**				€ 3,672	€ 10,238	€ 653	-	-	€ 7,738	€ 7,272	€ 4,408	-	-	€ 1,604	€ 3,719	€ 342	-	-	€ 1,040	€ 2,479	€ 77	**€ 521**	−€**2,589**	−€**564**	€ 11,421	€ 15,093
**Non-healthcare costs**																										
Paid home care	Hours	0.3	0.94	€ 90.3	€ 320	€ 0	0.9	1.01	€ 295	€ 343	€ 339	0.7	1.04	€ 240	€ 353	€ 0	0.7	1.04	€ 231	€ 353	€ 0	€ 205	−€55	−€9	€ 997	€ 1,087
Informal care	Hours	124	349	€ 3,371	€ 4,435	€ 1,386	134	195	€ 3,193	€ 2,984	€ 2,446	40.7	115	€ 1,047	€ 1,672	€ 440	38	1.27	€ 1,078	€ 1,404	€ 693	−€178	−€2,146	€ 31	€ 6,396	€ 9,767
Inability to perform unpaid labor	Day	1.7	4.13	€ 1,083	€ 1,814	€ 0	36.5	40.10	€ 22,489	€ 10,960	€ 24,968	23.7	34.70	€ 11,343	€ 12,399	€ 3,334	1.1	2.81	€ 696	€ 1,261	€ 0	€ 21,406	−€11,146	−€10,647	€ 35,224	€ 36,307
Productivity Losses	Day	1.6	2.50	€ 82.70	€ 150	€ 0	2	3	€ 101	€ 258	€ 0	1.8	2.67	€ 108	€ 238	€ 0	1.6	2.68	€ 64	€ 129	€ 0	€ 18	€ 7	−€44	€ 337	€ 419

**Total non-healthcare costs**				€ 4,627	€ 6,719	€ 1,386	-	-	€ 26,078	€ 14,545	€ 27,753	-	-	€ 12,738	€ 14,662	€ 3,774	-	-	€ 2,069	€ 3,147	€ 693	**€ 21,451**	−€**13,340**	−€**10,669**	€ 42,954	€ 47,581

**Total healthcare and productivity costs**				€ 8,299	€ 16,957	€ 2,039	-	-	€ 33,816	€ 21,817	€ 32,161	-	-	€ 14,342	€ 18,381	€ 4,116	-	-	€ 3,109	€ 5,626	€ 770	**€ 21,972**	**−€15,929**	**−€11,233**	€ 54,376	€ 62,675

GP, general practitioner; POH, practice nurse; physio, physiotherapist; occ, occupational therapist; speech, speech therapist; homeo, homeopath; psycho, psychologist; SEH, emergency room.

### Quality of Life, seizure frequency

**Fig. 2:**
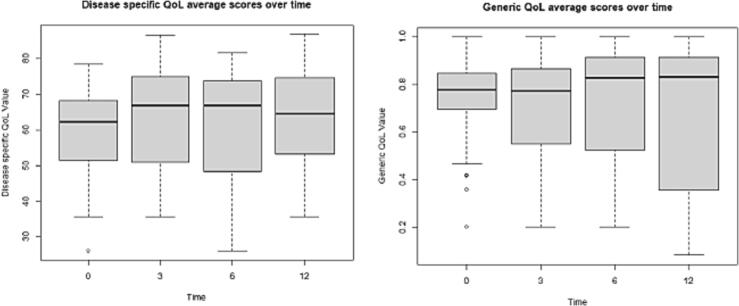
Boxplot disease-specific QoL average scores per time point (left) and boxplot generic health-related QoL average scores over time (right).

Fig. 2 presents the average disease-specific QoL and generic health-related QoL scores, which increased over time (59.3 to 64.1 and 0.78 to 0.82 respectively) however demonstrated no statistical significance (Table 3). Most generic health-related and disease-specific QoL subcategory scores increased over time (Supplementary Fig. 2, Supplementary Table 5, and Supplementary Fig. 3).

**Table 3:** Results of linear mixed models (1–3), logistic regressions (4–5), and linear regressions (4–29). The time points demonstrate the linear mixed model correlation of that time point with baseline (before surgery), the p value represents the statistical significance (set at 0.05), and the coefficient represents the change and direction of the correlation between the variable and time. The odds ratio illustrates the odds that the selected outcome will occur in the presence of undergoing surgery compared to the odds of the outcome occurring in the absence of the exposure to surgery. CI, confidence interval; OR, operation room; QoL, quality of life; Nr, number; ASM, anti-seizure medicine.

	Time point	Coefficient (CI)	P-value	Odds Ratio
1. Disease-specific QoL	3 months	3.69 (−2.59–9.98)	0.246	–
	6 months	3.33 (−2.96–9.61)	0.296	–
	12 months	4.81 (−1.48–11.10)	0.132	–
2. Generic QoL		−0.01 (−0.01–0.01)	0.416	–
3. Seizure frequency	3 months	−3.43 (−3.90– −2.97)	0.000	**–**
	6 months	−3.13 (−3.60– −2.67)	0.000	**–**
	12 months	−2.90 (−3.37– −2.43)	0.000	**–**
4. seizure freq + Disease-specific QoL	–	−0.02 (−0.03– −0.01)	0.037	0.50
5. seizure freq + Generic QoL	–	−0.15 (−0.99–0.69)	0.730	0.46
6. Disease-specific QoL + age	–	−0.15 (−0.39–0.-0.09)	0.212	–
7. Disease-specific QoL + age onset	–	−0.13 (−0.34––0.07)	0.202	–
8. Disease-specific QoL + duration	–	0.04 (−0.23–0.31)	0.756	–
9. Disease-specific QoL + gender	–	−2.74 (10.29 – 4.80)	0.463	–
10. Disease-specific QoL + education	–	0.65 (−1.27–2.57)	0.494	–
11. Disease-specific QoL + BMI	–	0.12 (−0.63–0.87)	0.748	–
12. Disease-specific QoL + comorbidities	–	−6.05 (−12.9–0.90)	0.085	–
13. Disease-specific QoL + neuromodulation	–	−2.97 (−17.32–11.38)	0.674	–
14. Disease-specific QoL + ketogenic diet	–	−7.77 (−27.55–12.01)	0.438	–
15. Disease-specific QoL + Nr of current ASMs	–	−0.77 (−2.95–1.41)	0.483	–
16. Disease-specific QoL + ASM history	–	−1.87 (−3.59- −0.15)	0.034	–
17. Disease-specific QoL + OR history	–	−3.52(−13.9–6.95)	0.497	–
18. Generic QoL + age	–	−0.001 (−0.01–0.01)	0.709	–
19. Generic QoL + age onset	–	−0.001 (−0.01–0.01)	0.778	–
20. Generic QoL + duration	–	0.0001 (−0.01–0.01)	0.960	–
21. Generic QoL + gender	–	−0.09 (−0.23–0.04)	0.153	–
22. Generic QoL + education	–	0.01 (−0.03–0.04)	0.687	–
23. Generic QoL + BMI	–	−0.01 (−0.02–0.01)	0.405	–
24. Generic QoL + comorbidities	–	−0.09 (−0.22–0.04)	0.169	–
25. Generic QoL + neuromodulation	–	0.02 (−0.25–0.28)	0.909	–
26. Generic QoL + ketogenic diet	–	−0.01 (−0.38–0.36)	0.974	–
27. Generic QoL + Nr of current ASMs	–	−0.01 (−0.05–0.03)	0.761	–
28. Generic QoL + ASM history	–	−0.01 (−0.04–0.02)	0.561	–
29. Generic QoL + OR history	–	0.14 (−0.04–0.33)	0.125	–

Across all time points, seizure frequency decreased in most patients after undergoing surgery compared to baseline (p-value < 0.000) (see Table 3). Patients whose seizure frequency decreased experienced a statistically higher disease-specific QoL score over time (p-value = 0.037). Moreover, patients who tried less ASM types in the past (ASM history) experienced a higher disease-specific QoL change after RES compared to patients who had an ASM history of ≥ 5 ASM (p-value = 0.034).

The significant results mentioned above combined with the relatively little change of the total scores of disease-specific QoL over time raised questions about the clinical significance of the disease-specific QoL scores. This suggested the need for further investigation to better understand the nuanced interplay between seizure frequency, QoL domains, and overall QoL in individual patients.

After 12 months, disease-specific non-responders consisted of 8 patients (27%), intermediate responders consisted of 7 patients (23%), and responders consisted of 15 patients (50%). In total, 73% of patients increased in their disease-specific QoL scores over time, where 50% reached a MCIC of 11.8 points or higher (Supplementary Fig. 4 and Supplementary Table 6). The MCIC for the generic health-related QoL scores was defined as 0.08. Generic health-related non-responders consisted of 11 patients (37%), intermediate responders consisted of 2 patients (6%), and responders consisted of 15 patients (57%) (Supplementary Fig. 5 and Supplementary Table 7). For both QoL measures, non- and intermediate-responders mainly consisted of patients who still suffered from seizures (ILAE class 2–5) and some who reached seizure freedom (ILAE class 1).

In total, seizure frequency non-responders consisted of 2 patients, intermediate responders consisted of 4 patients, and responders consisted of 23 patients. Before surgery, 13% of patients reported having 4 to 12 seizure days annually and 83% of patients reported daily seizures (Supplementary Fig. 6). From 3 months to 12 months after surgery, in total 4 patients lost their seizure freedom and shifted from ILAE class 1 to a higher classification, leading to 73% of patients maintaining seizure freedom 12 months after RES.

## Discussion

### Economic and clinical outcomes

To our knowledge, this is the first study that offers an overview of the SC of RES and provides an analysis covering both economic and disease-specific clinical outcomes. Our study demonstrates that the average SC of one patient from 3 months prior to surgery until the first year post-surgery entailed €54,376 and decreases over time. A French study of 2016 confirms that total costs-of-disease decrease after RES and that the procedure is safe, effective, and cost-saving [25]. It is important to acknowledge the comparison of the SC presented in this study that entailed the first year after RES to annual care-as-usual SC in a similar patient population. A Dutch study from 2014 investigating SC for 203 DRE patients on ASM, including costs of possible side effects, estimated the SC per patient to be €20,751 annually [16]. Our results show that the first year after RES is higher SC per patient compared to these annual SC of ASM treatment. Nevertheless, our study presents valuable information on the trend of SC after RES.

Our clinical outcomes, namely 50% of patients increasing in disease-specific QoL, 53% of patients increasing in generic health-related QoL, and 73% of patients maintaining seizure freedom, add onto the current body of literature. For instance, a randomized controlled trial demonstrated that care-as-usual led to lower and less increase in QoL in comparison to patients undergoing RES [11]. Moreover, prospective cohort studies demonstrated that disease-specific QoL increases in 58–61% of patients undergoing RES [11], [12], [44]. Postoperative seizure frequency decrease after RES also corresponds with findings of others, who have reported seizure freedom in 50–68% of surgical candidates [17], [45]. Lastly, our result which shows that seizure reduction was associated with an increase in disease-specific QoL is in line with several studies with the same research design [21], [22], [46].

### Limitations

Firstly, the baseline measurement of QoL in our study may have been over- or underestimated, due to feelings of either hope or anxiety (or both). In addition, the relatively minimal change observed in the average total QoL scores could be attributed to the potential nullification of individual QoL scores. More specifically, the improvements in QoL for some patients may be counterbalanced by deteriorations or limited improvements in others. It is therefore imperative to place emphasis on the clinical significance of QoL change via MCIC value comparisons. As a result, a deeper insight could be provided into the effectiveness of RES and the overall enhancement of QoL in patients undergoing RES and thereby refine the understanding of the nuanced factors influencing QoL in this specific patient population.

Secondly, there was a discrepancy in the interpretations of one question regarding type of medical consumption received. Whilst patients reported to not have received any medical care, patient’s files reported differently. Patients were probably not fully informed on their treatment during their visits or misinterpreted the meaning of the question. Therefore, it was decided to solely rely on the cross-check of the patient files to prevent incomplete data. Although this increased the accuracy of results for regional patients, some information on patients coming from other regions could still have been missed. Nonetheless, we believe this method is more accurate than solely relying on patients’ answers. Although power was reached and study design was accounted for, other limitations include the limited sample size and absence of a comparator group, where the latter could offer the ability to perform cost-effectiveness analyses.

Finally, baseline was set shortly before surgery and referred to the 3 months prior to surgery as our objective was to study the costs associated with RES. We have chosen this period because within these 3 months, patients go through the process to prepare for surgery including meeting the neurosurgeon, anesthesiologist, and epilepsy nurse. This means the costs of diagnostic evaluation including diagnostic MRI, video-EEG, and other diagnostic/presurgical evaluations were not taken into account. It would be interesting to analyze these costs in future projects; those should then be aimed at analyzing the cost-effectiveness of referral for diagnostic/presurgical evaluation, also taking into account the patients not identified as candidates for resective brain surgery.

### Future research

Several steps should be taken to maintain momentum in this field of research. Firstly, by incorporating an additional component of caregivers’ perspective. This could help create a better representation on QoL and productivity losses of caregivers, the effect epilepsy has on the patients’ environment, and the added costs that accompany epilepsy as a disease to a broader extent.

Secondly, although already set in motion by our research team, this research should be extended to a larger sample size with a longer follow-up period, to incorporate for instance the effect of decreasing use of ASM and further analyze the trend in SC after RES. Thirdly, the complete diagnostic/presurgical evaluation and care-as-usual group as comparator should be included as well to create a more comprehensive picture of patient costs. Lastly, by incorporating expectations of patients before agreeing to surgery, future research could offer a clear patient profile of DRE patients who would benefit most from RES.

When concentrating on the Dutch population, +/− 130 patients undergo RES annually, while 2100 patients are expected to be surgical candidates based on fact that 20–50% of DRE patients are eligible for RES [9]. This confirms the findings from peer-reviewed studies that RES is currently underutilized [2], [3], [4]. Underutilization may be explained by the lack of complete economic studies showing the direct and indirect economic benefits which are becoming increasingly important for societies. Since healthcare costs are rising considerably and are expected to rise more in the near future, the Dutch government is becoming increasingly critical concerning reimbursement of costly treatments [47]. As DRE patients who are not surgically treated generally continue to be patients and actively consume health care, lifetime costs insinuate no decrease [21], [24]. Therefore, a study assessing the complete diagnostic/presurgical evaluation, SC, and disease-specific QoL in the same population could provide relevant information for policy makers and researchers [47].

## Conclusion

This study presents evidence for impact on SC and disease-specific and generic health-related QoL in the first 12 months after RES in patients with DRE. As DRE patients are committed to lifelong reduced QoL, lifelong ASM use, and yearly SC of €20,751, future research on RES should encompass longer follow-up periods, larger sample size, and a cost-effectiveness analysis with a comparator. Overall, this study reports on the SC directly related to RES and could serve as a pilot project to offer an up-to-date model for larger cost-effectiveness studies in the future.

## Authors' contributions

All authors contributed to the study design, interpretation of findings and writing of the manuscript. All authors approved the final version of the manuscript for submission.

## Statements and Declarations: Competing interests and funding

Mickaël Hiligsmann has received research grants paid to his institution by Amgen, Radius Health and Angelini Pharma, advisory grant from Pfizer (paid to institution), lecture fees (paid to institution) from IBSA, all outside the current work. All other authors declare no conflicts of interest. This study was not funded.

## Declaration of Competing Interest

The authors declare that they have no known competing financial interests or personal relationships that could have appeared to influence the work reported in this paper.
