# Supplementary material for: Societal costs and quality of life analysis in patients undergoing resective epilepsy surgery: A one-year follow-up
Source: Epilepsy Behav Rep. 2023 Nov 19;24:100635. doi: 10.1016/j.ebr.2023.100635 (PMC10711171; doi:10.1016/j.ebr.2023.100635)
Supplement: Supplementary data 1 [file mmc1.docx]

# Appendix

Supplementary Table 1 & 2

STROBE Statement—Checklist of items that should be included in reports of ***cohort studies***

|  | Item No | Recommendation | Page No |
| --- | --- | --- | --- |
| **Title and abstract** | 1 | (*a*) Indicate the study’s design with a commonly used term in the title or the abstract |  |
|  |  | (*b*) Provide in the abstract an informative and balanced summary of what was done and what was found | 1-2 |
| Introduction | | | |
| Background/rationale | 2 | Explain the scientific background and rationale for the investigation being reported | 3 |
| Objectives | 3 | State specific objectives, including any prespecified hypotheses | 4 |
| Methods | | | |
| Study design | 4 | Present key elements of study design early in the paper | 5 |
| Setting | 5 | Describe the setting, locations, and relevant dates, including periods of recruitment, exposure, follow-up, and data collection | 5 |
| Participants | 6 | (*a*) Give the eligibility criteria, and the sources and methods of selection of participants. Describe methods of follow-up | 5 |
|  |  | (*b*) For matched studies, give matching criteria and number of exposed and unexposed |  |
| Variables | 7 | Clearly define all outcomes, exposures, predictors, potential confounders, and effect modifiers. Give diagnostic criteria, if applicable | 6 |
| Data sources/ measurement | 8* | For each variable of interest, give sources of data and details of methods of assessment (measurement). Describe comparability of assessment methods if there is more than one group | 6 |
| Bias | 9 | Describe any efforts to address potential sources of bias | 5 |
| Study size | 10 | Explain how the study size was arrived at | 5 |
| Quantitative variables | 11 | Explain how quantitative variables were handled in the analyses. If applicable, describe which groupings were chosen and why | 6-7 |
| Statistical methods | 12 | (*a*) Describe all statistical methods, including those used to control for confounding | 7 |
|  |  | (*b*) Describe any methods used to examine subgroups and interactions |  |
|  |  | (*c*) Explain how missing data were addressed |  |
|  |  | (*d*) If applicable, explain how loss to follow-up was addressed |  |
|  |  | (*e*) Describe any sensitivity analyses |  |
| Results | | |  |
| Participants | 13* | (a) Report numbers of individuals at each stage of study—eg numbers potentially eligible, examined for eligibility, confirmed eligible, included in the study, completing follow-up, and analysed | 8 |
|  |  | (b) Give reasons for non-participation at each stage |  |
|  |  | (c) Consider use of a flow diagram |  |
| Descriptive data | 14* | (a) Give characteristics of study participants (eg demographic, clinical, social) and information on exposures and potential confounders | 8 |
|  |  | (b) Indicate number of participants with missing data for each variable of interest |  |
|  |  | (c) Summarise follow-up time (eg, average and total amount) |  |
| Outcome data | 15* | Report numbers of outcome events or summary measures over time | 9 |

| Main results | 16 | (*a*) Give unadjusted estimates and, if applicable, confounder-adjusted estimates and their precision (eg, 95% confidence interval). Make clear which confounders were adjusted for and why they were included | 9 |
| --- | --- | --- | --- |
|  |  | (*b*) Report category boundaries when continuous variables were categorized |  |
|  |  | (*c*) If relevant, consider translating estimates of relative risk into absolute risk for a meaningful time period |  |
| Other analyses | 17 | Report other analyses done—eg analyses of subgroups and interactions, and sensitivity analyses | 11-12 |
| Discussion | | | |
| Key results | 18 | Summarise key results with reference to study objectives | 16 |
| Limitations | 19 | Discuss limitations of the study, taking into account sources of potential bias or imprecision. Discuss both direction and magnitude of any potential bias | 17 |
| Interpretation | 20 | Give a cautious overall interpretation of results considering objectives, limitations, multiplicity of analyses, results from similar studies, and other relevant evidence | 17 |
| Generalisability | 21 | Discuss the generalisability (external validity) of the study results | 18 |
| Other information | | | |
| Funding | 22 | Give the source of funding and the role of the funders for the present study and, if applicable, for the original study on which the present article is based | 2 |

*Give information separately for exposed and unexposed groups.

**Note:** An Explanation and Elaboration article discusses each checklist item and gives methodological background and published examples of transparent reporting. The STROBE checklist is best used in conjunction with this article (freely available on the Web sites of PLoS Medicine at http://www.plosmedicine.org/, Annals of Internal Medicine at http://www.annals.org/, and Epidemiology at http://www.epidem.com/). Information on the STROBE Initiative is available at http://www.strobe-statement.org.

CHEERS 2022 Checklist

| **Topic** | **No.** | **Item** | **Location where item is reported** |
| --- | --- | --- | --- |
| **Title** |  |  |  |
|  | 1 | Identify the study as an economic evaluation and specify the interventions being compared. | 1 |
| **Abstract** |  |  |  |
|  | 2 | Provide a structured summary that highlights context, key methods, results, and alternative analyses. | 2 |
| **Introduction** |  |  |  |
| **Background and objectives** | 3 | Give the context for the study, the study question, and its practical relevance for decision making in policy or practice. | 3 |
| **Methods** |  |  |  |
| **Health economic analysis plan** | 4 | Indicate whether a health economic analysis plan was developed and where available. | 5 |
| **Study population** | 5 | Describe characteristics of the study population (such as age range, demographics, socioeconomic, or clinical characteristics). | 5 |
| **Setting and location** | 6 | Provide relevant contextual information that may influence findings. | 5 |
| **Comparators** | 7 | Describe the interventions or strategies being compared and why chosen. | 5 |
| **Perspective** | 8 | State the perspective(s) adopted by the study and why chosen. | 5 |
| **Time horizon** | 9 | State the time horizon for the study and why appropriate. | 5 |
| **Discount rate** | 10 | Report the discount rate(s) and reason chosen. | 6 |
| **Selection of outcomes** | 11 | Describe what outcomes were used as the measure(s) of benefit(s) and harm(s). | 6 |
| **Measurement of outcomes** | 12 | Describe how outcomes used to capture benefit(s) and harm(s) were measured. | 6 |
| **Valuation of outcomes** | 13 | Describe the population and methods used to measure and value outcomes. | 6 |
| **Measurement and valuation of resources and costs** | 14 | Describe how costs were valued. | 6 |
| **Currency, price date, and conversion** | 15 | Report the dates of the estimated resource quantities and unit costs, plus the currency and year of conversion. | 6 |
| **Rationale and description of model** | 16 | If modelling is used, describe in detail and why used. Report if the model is publicly available and where it can be accessed. | - |
| **Analytics and assumptions** | 17 | Describe any methods for analysing or statistically transforming data, any extrapolation methods, and approaches for validating any model used. | 7 |
| **Characterising heterogeneity** | 18 | Describe any methods used for estimating how the results of the study vary for subgroups. | 7 |
| **Characterising distributional effects** | 19 | Describe how impacts are distributed across different individuals or adjustments made to reflect priority populations. | 5 |
| **Characterising uncertainty** | 20 | Describe methods to characterise any sources of uncertainty in the analysis. | 7 |
| **Approach to engagement with patients and others affected by the study** | 21 | Describe any approaches to engage patients or service recipients, the general public, communities, or stakeholders (such as clinicians or payers) in the design of the study. | 5 |
| **Results** |  |  |  |
| **Study parameters** | 22 | Report all analytic inputs (such as values, ranges, references) including uncertainty or distributional assumptions. | 8 |
| **Summary of main results** | 23 | Report the mean values for the main categories of costs and outcomes of interest and summarise them in the most appropriate overall measure. | 8 |
| **Effect of uncertainty** | 24 | Describe how uncertainty about analytic judgments, inputs, or projections affect findings. Report the effect of choice of discount rate and time horizon, if applicable. | - |
| **Effect of engagement with patients and others affected by the study** | 25 | Report on any difference patient/service recipient, general public, community, or stakeholder involvement made to the approach or findings of the study | 8 |
| **Discussion** |  |  |  |
| **Study findings, limitations, generalisability, and current knowledge** | 26 | Report key findings, limitations, ethical or equity considerations not captured, and how these could affect patients, policy, or practice. | 16 |
| **Other relevant information** |  |  |  |
| **Source of funding** | 27 | Describe how the study was funded and any role of the funder in the identification, design, conduct, and reporting of the analysis | 2 |
| **Conflicts of interest** | 28 | Report authors conflicts of interest according to journal or International Committee of Medical Journal Editors requirements. | 2 |

*From:* Husereau D, Drummond M, Augustovski F, et al. Consolidated Health Economic Evaluation Reporting Standards 2022 (CHEERS 2022) Explanation and Elaboration: A Report of the ISPOR CHEERS II Good Practices Task Force. Value Health 2022;25. <doi:10.1016/j.jval.2021.10.008>

Supplementary Table 3

Table 3: Unit prices for healthcare and non-healthcare costs

| *Costs (category)* | *Unit* | *Cost per unit (Euros)* |
| --- | --- | --- |
| *Healthcare* |  |  |
| General practitioner | Contact | *66* |
| Practice nurse | Contact | *17* |
| Social worker | Contact | *65* |
| Physiotherapist | Contact | *333* |
| Occupational therapist | contact | *33* |
| Speech therapist | contact | *30* |
| Dietician | contact | *72* |
| Homeopath | Contact | *105* |
| Psychologist | Contact | *64* |
| Occupational physician | Contact | *338,75* |
| Emergency room | Contact | *259* |
| Ambulance | contact | *515* |
| Epileptic institution | Visit | *153* |
| Epileptic institution | Night | *460* |
| Hospitalization (neurology) | Days | *395* |
| Medical specialist | Contact | *163* |
| Diagnostic treatment: MRI scan | - | *206* |
| Diagnostic treatment: CT scan | - | *129* |
| Diagnostic treatment: Echography scan | - | *88* |
| Medication | - | *-* |
| Non-healthcare |  |  |
| Paid home care | Hours | *73* |
| Informal care household tasks | Hours | *23* |
| Informal care Personal care | Hours | *50* |
| Informal care | Hours | *73* |
| Inability to perform unpaid labor | Day | *14* |
| Productivity losses | Day | *31,60 (female)*  *37,90 (male)* |

Supplementary Figure 1


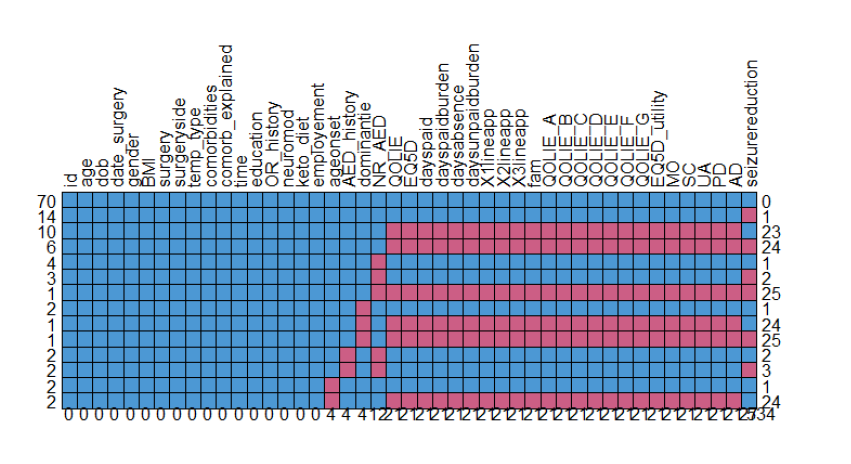


*Figure 1: Missing data overview. In pink missing observations are represented, in blue the complete observations are represented. On the x axis the variables are stated, the y axis represents the number of missing cases, and the z axis represents the number of complete cases.*

Supplementary Table 4

Table 4: Demographic characteristics of employed and unemployed patients. Continuous variables are presented using the mean and standard deviation (SD) and binomial variables are presented in frequencies (freq), categorical variables with more than two categories are presented as frequencies and percentages. Freq, frequency; OR, operation room.

|  | **Employed(n=17)** | **Non-employed (n=13)** |
| --- | --- | --- |
| Duration mean | 17 years | 14 years |
| Age mean | 44 years | 45 years |
| Age onset mean | 24 years | 28 years |
| Dominance | 9 (53%) | 7 (54%) |
| Gender: female | 6 (35%) | 3 (23%) |
| OR history | 3 (18%) | 2 (15%) |
| Surgery side: left | 7 (41%) | 5 (38%) |
| Education range | 2-9 | 2-9 |
| Comorbidities | 5 (29%) | 9 (69%) |

Supplementary Figure 2


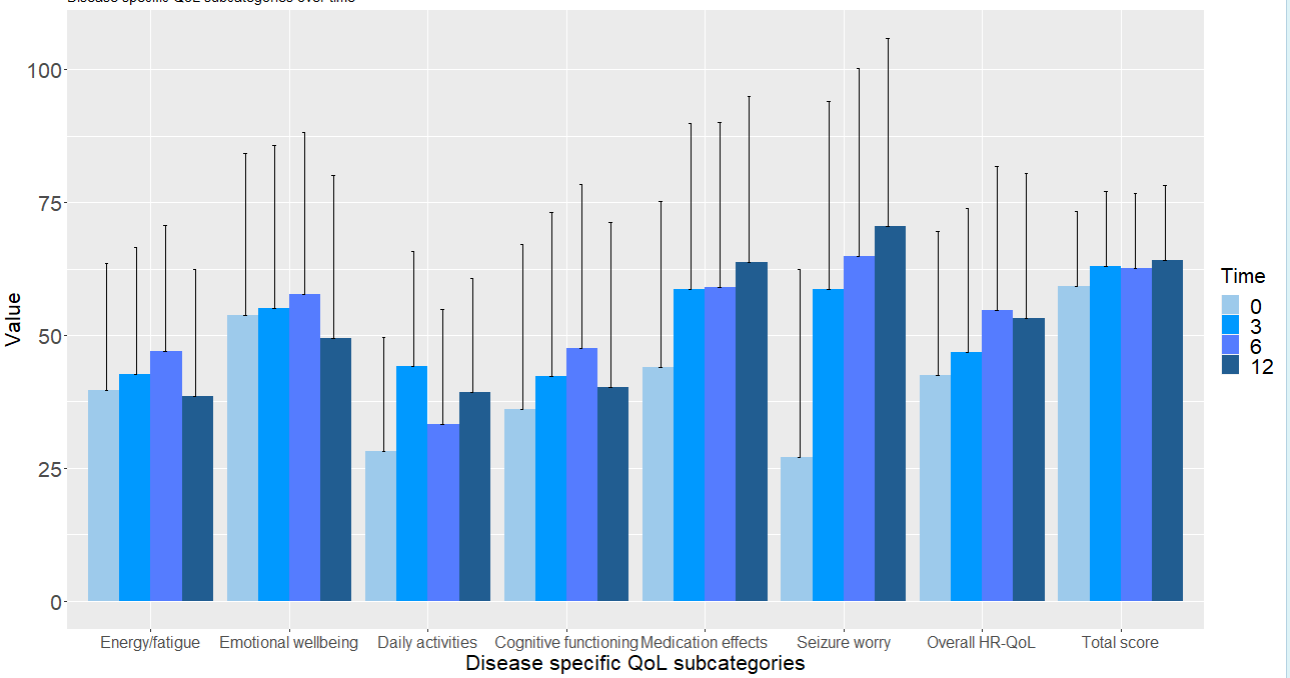


Figure 2: disease-specific QoL subscores over time with their standard deviation as error bars. Error bars are identical in value in both directions (positive and negative). QoL, quality of life.

Supplementary Table 5:

Table 5: Average disease-specific QoL (QOLIE-31P) scores and standard deviations of subcategories

| Sub category | Time point | QOLIE-31P sub score | SD |
| --- | --- | --- | --- |
| Energy/fatigue | 0 | 39.66667 | 23.86108 |
|  | 3 | 42.62500 | 23.86108 |
|  | 6 | 46.91667 | 23.86108 |
|  | 12 | 38.50000 | 23.86108 |
| Emotional wellbeing | 0 | 53.72667 | 30.58488 |
|  | 3 | 55.15333 | 30.58488 |
|  | 6 | 57.72667 | 30.58488 |
|  | 12 | 49.52000 | 30.58488 |
| Daily activities | 0 | 28.11500 | 21.52074 |
|  | 3 | 44.20167 | 21.52074 |
|  | 6 | 33.29833 | 21.52074 |
|  | 12 | 39.24500 | 21.52074 |
| Cognitive functioning | 0 | 36.11875 | 30.99897 |
|  | 3 | 42.21792 | 30.99897 |
|  | 6 | 47.48850 | 30.99897 |
|  | 12 | 40.18808 | 30.99897 |
| Medication effects | 0 | 44.04893 | 31.20558 |
|  | 3 | 58.74984 | 31.20558 |
|  | 6 | 58.98293 | 31.20558 |
|  | 12 | 63.83728 | 31.20558 |
| Seizure worry | 0 | 27.08373 | 35.30895 |
|  | 3 | 58.69017 | 35.30895 |
|  | 6 | 64.94617 | 35.30895 |
|  | 12 | 70.58267 | 35.30895 |
| Overall HR-QoL | 0 | 42.44167 | 27.23384 |
|  | 3 | 46.72917 | 27.23384 |
|  | 6 | 54.62950 | 27.23384 |
|  | 12 | 53.22917 | 27.23384 |
| Total score | 0 | 59.28054 | 14.13911 |
|  | 3 | 62.97254 | 14.13911 |
|  | 6 | 62.60810 | 14.13911 |
|  | 12 | 64.09173 | 14.13911 |

Supplementary Figure 3


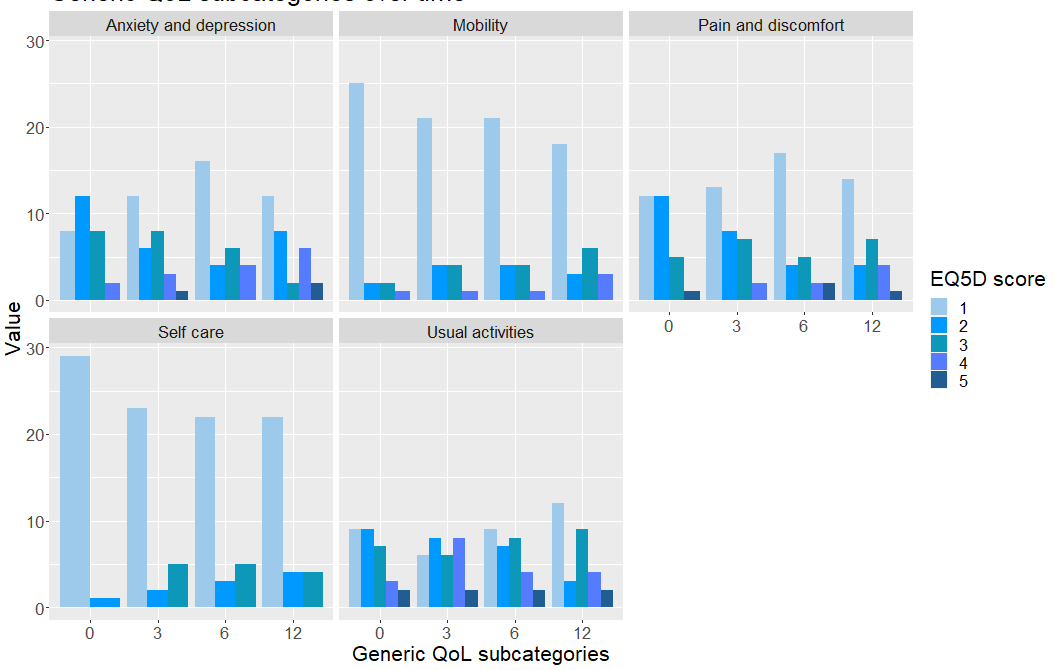


Figure 3: generic QoL subscores before and three-, six-, and twelve-months after surgery. Results are presented as patient frequencies (n=30) and their scores per time point. QoL, quality of life; EQ5D, 5-dimension health-related quality of life instrument

Supplementary Figures 4 & 5


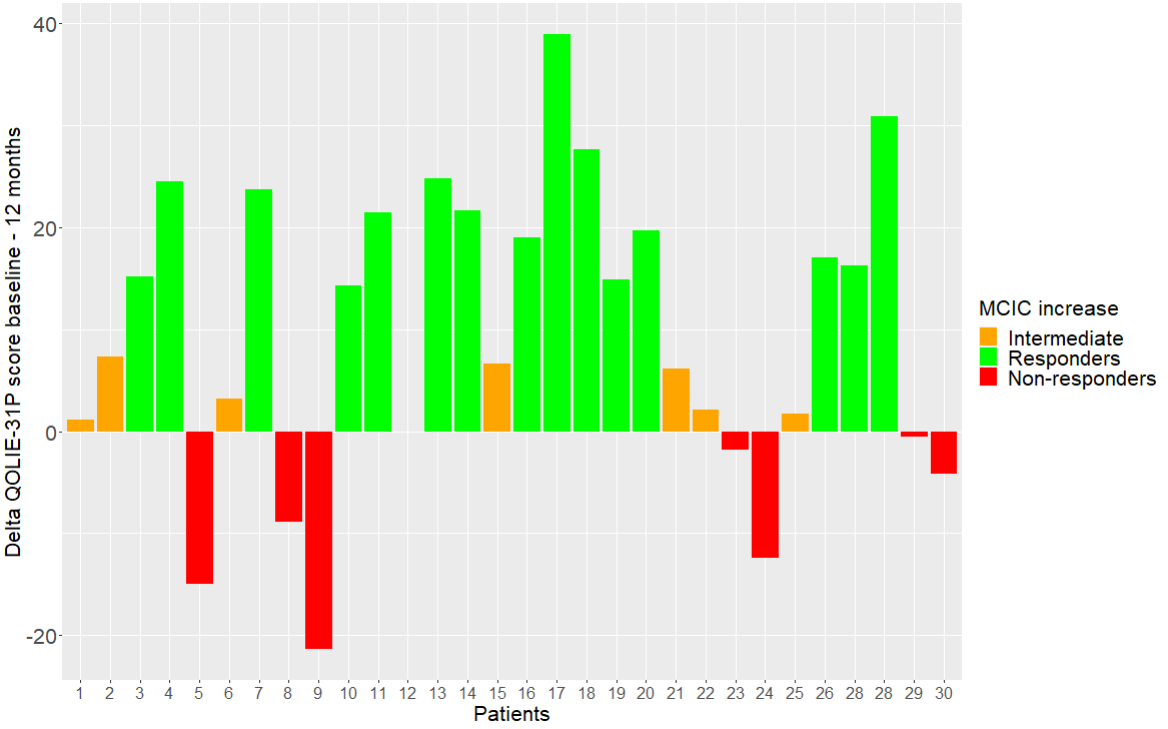


Figure 4: Delta disease-related QoL utility score of baseline (pre-surgery) and post-surgery (6 or 12 months). Patients either increase equal to or higher than 0.08 (green), increase but not equal to or higher than 0.08 (orange), or decrease in their utility score (red). QoL, quality of life. MCIC=minimal clinically important change


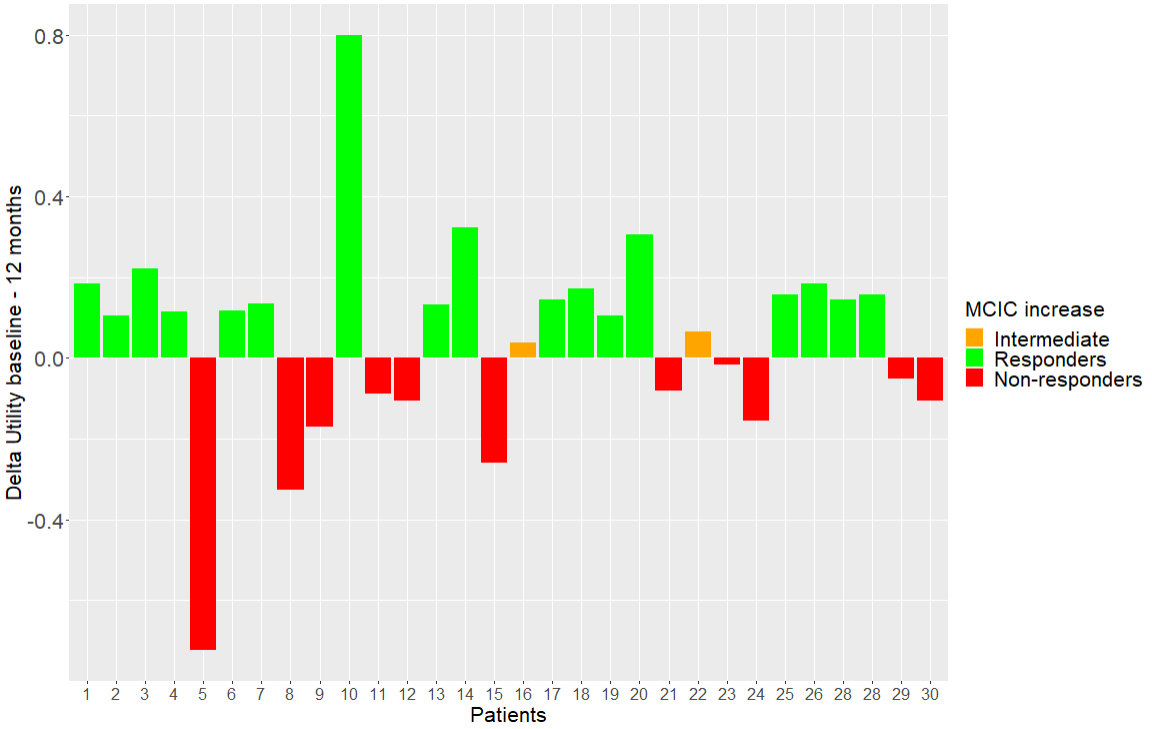


Figure 5: Delta generic health-related QoL utility score of baseline (pre-surgery) and post-surgery (6 or 12 months). Patients either increase equal to or higher than 0.08 (green), increase but not equal to or higher than 0.08 (orange), or decrease in their utility score (red). QoL, quality of life; MCIC, minimal clinically important change.

Supplementary Table 6 & 7

Table 6: Distinctive baseline demographics per disease-specific QoL group. Continuous variables are presented using the mean and standard deviation (SD) and binomial variables are presented in frequencies (freq), categorical variables with more than two categories are presented as frequencies and percentages. QoL, quality of life; SD, standard deviation; AED, anti-epileptic drug; OR, operation room; ATL, anterior temporal lobectomy; AH, amygdalohippocampectomy; w/o, without.

| Variables | Disease-specific QoL group | | |
| --- | --- | --- | --- |
|  | Responder (n=15) | Intermediate responder (n=7) | Non-responder (n=8) |
| Age (SD) | 44.7 (13.0) | 41.3 (8.2) | 45.1 (20.0) |
| Age onset (SD) | 22.4 (16.6) | 30.9 (4.85) | 26.3 (21.0) |
| Duration (SD) | 19.9 (12.8) | 7.9 (6.87) | 16.7 (14.0) |
| Dominance | 8 (53%) | 3 (43%) | 4 (50%) |
| Gender: female | 10 (67%) | 6 (86%) | 4 (50%) |
| OR history | 3 (20%) | 0 | 1 (12.5%) |
| Surgery side: left | 5 (30%) | 4 (57%) | 3 (37.5%) |
| Education range | Elementary school – other education | Hbo, hts, heao, hheo - University | Elementary school – University |
| Comorbidities | 4 (27%) | 4 (57%) | 4 (50%) |
| AED history: +5 | 7 (47%) | 4 (57%) | 2 (25%) |
| Temporal lobe resection type | 9 ATL with AH (60%)  1 extra temp (7%)  1 resection with temporal lesion (7%)  4 selective AH (27%) | 2 ATL with AH (29%)  5 selective AH (71%) | 3 ATL with AH (37.5%)  1 ATL w/o AH (12.5%)  1 extra temporal (12.5%)  4 selective AH (50%) |

Table 7: demographic characteristics of patients with increased utility score (green), increased but not higher than the Minimally Clinically Important Change (MCIC) (orange), and those that decreased in generic QoL utility score. Continuous variables are presented using the mean and standard deviation (SD) and binomial variables are presented in frequencies (freq), categorical variables with more than two categories are presented as frequencies and percentages. QoL, quality of life; SD, standard deviation; AED, anti-epileptic drug; OR, operation room; ATL, anterior temporal lobectomy; AH, amygdalohippocampectomy; w/o, without.

| Variables | Generic QoL group | | |
| --- | --- | --- | --- |
|  | Responder (n=17) | Intermediate responder (n=2) | Non-responder (n=11) |
| Age (SD) | 42.9 (11.4) | 36.5 (3.74) | 47 (18.6) |
| Age onset (SD) | 22.1 (13.7) | 29.5 (0.5) | 29.9 (20.3) |
| Duration (SD) | 18.3 (12.7) | 4.95 (3.58) | 19.9 (13.2) |
| Dominance | 8 (47%) | 2 (100%) | 5 (45.5%) |
| Gender: female | 5 (29%) | 1 (50%) | 4 (36%) |
| OR history | 3 (17.5%) | 0 | 1 (9%) |
| Surgery side: left | 6 (35%) | 1 (50%) | 5 (45.5%) |
| Education range | Elementary school – other education | Mbo/mts/meao/mhno –Hbo,hts,heao, hhno | Elementary school – University |
| Comorbidities | 5 (29%) | 1 (50%) | 6 (55%) |
| AED history: +5 | 3 (17.5%) | 0 | 4 (36%) |
| Temp type | 9 ATL with AH (53%)  1 extra temporal (6%)  1 resection with temporal lesion (6%)  6 selective AH (35%) | 2 selective ah (100%) | 5 ATL with AH (45.5%)  1 ATL w/o AH (9%)  1 extra temporal (9%)  4 selective AH (36%) |

Supplementary Figure 6


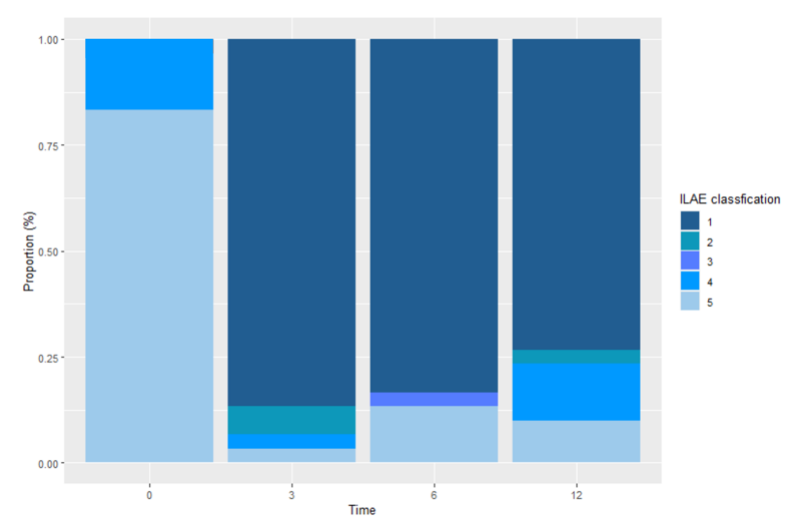


Figure 6: preoperative seizure frequency (where light blue refers to patients reporting daily seizures and coral blue refers to patients reporting four to twelve seizure days annually) and its postoperative ILAE classification per time point. ILAE, International League Against Epilepsy.
